# Establishment and characterization of an immortalized bovine luteal cell line

**DOI:** 10.3389/fvets.2026.1758622

**Published:** 2026-05-18

**Authors:** Guoqing Fei, Zefang Zhao, Minghua Zeng, Yue Liang, Ting Miao, Yanqiu Liu, Jiayao Yang, Shulin Chen, Xiaoyan Zhu, Jianguo Wang

**Affiliations:** 1 College of Veterinary Medicine, Northwest A&F University, Yangling, China; 2 Key Laboratory of Shaanxi Universities, Hanzhong Vocational and Technical College, Hanzhong, China; 3 Hanzhong Key Laboratory of Clinical Molecular Biology, Hanzhong Vocational and Technical College, Hanzhong, China

**Keywords:** bovine luteal cell, co-culture, immortalization, steroidogenesis, SV40 T antigen

## Abstract

The bovine corpus luteum (CL) is critical for pregnancy establishment and maintenance via progesterone (P_4_) secretion. Primary bovine luteal cells (PBLC), the core *in vitro* model for reproductive and steroidogenesis research, are severely limited by high isolation cost, cellular heterogeneity, and restricted proliferative capacity. In this study, we established an immortalized bovine luteal cell line with stable *in vitro* proliferative capacity, termed SV40 T-immortalized bovine luteal cells (SV40T-IBLC), via lentiviral transduction of the Simian virus 40 (SV40) T antigen gene. The immortalized cells displayed a typical epithelial-like morphology with abundant small cytoplasmic lipid droplets. They secreted multiple functional hormones including P_4_ and oxytocin, expressed synaptophysin, a key marker protein of mature functional luteal cells, and maintained stable expression of core steroidogenic genes and proteins such as STAR, 3β-hydroxysteroid dehydrogenase (3β-HSD), CYP11A1, and PTGFR, with mRNA levels of these key genes showing no significant difference from those of parallel-cultured PBLC. Functionally, SV40T-IBLC exhibited time-dependent P_4_ secretion, with P_4_ concentration in the culture supernatant reaching 1.89 ± 0.14 ng/mL at 60 h, which was fully consistent with the secretory level and dynamic pattern of PBLC. After continuous *in vitro* culture up to the 50th passage, the cells maintained a normal diploid karyotype (29 pairs of autosomes and 1 pair of XX sex chromosomes), and no evidence of malignant transformation was observed in the anchorage-independent growth assay and *in vivo* tumorigenicity assay in nude mice. Furthermore, Co-culture with SV40T-IBLC significantly enhanced the viability of bovine endometrial epithelial cells (BEEC) by 34.2% after 72 h of co-incubation (*p* < 0.01), increased intracellular lipid droplet abundance of BEEC, and upregulated the mRNA expression of endometrial receptivity-related genes including EGF and LIF, with no significant alteration in BEEC apoptosis rate. In conclusion, this immortalized cell line retains core functional characteristics of PBLC, providing a stable, homogeneous *in vitro* model for bovine reproductive research.

## Graphical abstract

**Figure:**
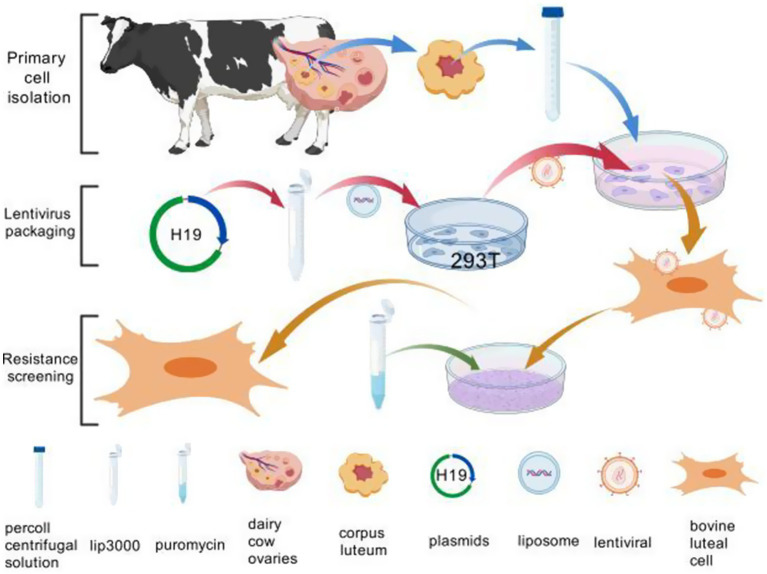

## 1 Introduction

The corpus luteum (CL) is a transient endocrine gland that appears cyclically on the ovary and undergoes a short but dynamic developmental process, including functional establishment and regression. Its proper function plays a central role not only in regulating the estrous cycle but also in determining embryo implantation and pregnancy maintenance (1, 2). During pregnancy recognition and embryo attachment in dairy cows, the CL regulates endometrial synchronization and inhibits uterine muscle peristalsis by secreting P_4_ and other cytokines, thereby creating favorable conditions for successful embryo attachment (3). Desynchronization of embryonic and maternal regulation during pregnancy establishment can lead to embryonic loss and reduced fertility. The elevated levels of luteal P_4_ synthesis can establish a stable pregnancy environment conducive to embryo survival. It has been reported that injection of luteinizing hormone (LH) in cattle can significantly promote luteal P_4_ synthesis and increase the local P_4_ concentration in the ovary and uterus, thus improving the pregnancy rate (4). Endometrial tolerance is another key factor affecting the establishment of pregnancy, and the CL directly regulates endometrial cell tolerance through endocrine regulation, providing essential conditions for embryo implantation (5, 6). Furthermore, Co-culture experiments of luteal cells with early embryos demonstrated that luteal cells exert a direct nourishing effect on early embryos, markedly improving embryo survival rates (7, 8). Taken together, these findings highlight the pivotal role of the CL in reproduction.

The secretion of steroid hormones by the CL is mainly controlled by large luteal cells (LLC) and small luteal cells (SLC) (9). Understanding how these cell populations regulate P_4_ synthesis is critical for advancing bovine reproductive efficiency (10, 11). Luteal cells are not only responsible for the majority of basal P_4_ synthesis but also serve as primary targets of interferon-*τ* (IFN-τ), which regulates pregnancy maintenance through the STAT1-IRF9 signaling pathway. Thus, luteal cells provide a representative *in vitro* model for investigating the regulatory mechanisms underlying CL function (12, 13). However, the application of PBLC is severely constrained by high isolation costs, heterogeneity, poor proliferative capacity, and limited number of passages, which restricts cellular and molecular investigations into CL biology (14, 15). Therefore, the establishment of an immortalized bovine luteal cell line has practical application value for further revealing the physiology of reproduction and optimizing *in vitro* embryo culture systems.

Telomerase reverse transcriptase (TERT) and Simian virus 40 (SV40) T antigen are widely used to construct immortalized cell lines. hTERT is generally thought to preserve the original biological properties of the cells, whereas SV40 T antigen enhances the cell proliferation (16–19). However, the application scope of these two immortalization strategies is strictly limited by the intrinsic biological characteristics of target cells. Specifically, hTERT-mediated immortalization is only applicable to cells that proliferate in a telomerase-dependent manner, and it cannot overcome replicative senescence in terminally differentiated cells with undetectable endogenous telomerase activity (20, 21). Bovine luteal cells (BLCs) are typical terminally differentiated steroidogenic cells, and accumulating evidence has confirmed that no telomerase activity is detectable in BLCs, whose telomere maintenance relies on alternative lengthening of telomeres rather than telomerase-mediated reverse addition (21, 22). In contrast, SV40 T antigen can efficiently break through the proliferation limit of primary BLCs: it binds and inactivates the key tumor suppressor proteins p53 and pRb, thereby blocking the two core cellular senescence pathways, releasing cell cycle arrest, and driving quiescent terminally differentiated cells to re-enter the cell cycle for continuous proliferation (23, 24). This mechanism gives SV40 T antigen a unique advantage in establishing immortalized cell lines from terminally differentiated cells, with well-documented high transformation efficiency and stable technical performance in multiple mammalian species (18, 19). Over the past decade, immortalized luteal cell lines have been successfully established in rats, goats, and pigs (25–27). However, no immortalized luteal cell line has yet been reported in cattle (28, 29). Although certain aspects of luteal function are conserved across mammals, substantial interspecies differences in reproductive physiology still exist (30, 31). In the present study, we hypothesized that lentiviral-mediated transduction of the SV40 T antigen could immortalize BLCs, creating a cell line that maintains the fundamental biological characteristics of primary bovine luteal cells (PBLCs) without malignant transformation. The main goal of this study was to establish and characterize an immortalized bovine luteal cell line and to verify its potential as an *in vitro* model for research on bovine reproductive physiology.

## 2 Materials and methods

### 2.1 Isolation, purification and culture of primary bovine luteal cells

Healthy bovine ovaries from early gestation (70–100 days of gestation; fetal crown-rump length < 15 cm) were collected from a slaughterhouse in Xi’an, China (32). They were preserved in ice-cold phosphate-buffered saline (PBS, Solarbio, Beijing, China) containing 100 IU/mL penicillin and streptomycin (Sigma-Aldrich, St. Louis, MO, United States), and transported promptly to the laboratory. The tissues were then transferred to an ultra-clean table for manipulation. The CL tissues were aseptically stripped and minced into fragments with a volume of ≤ 1 mm^3^, followed by rinsing with PBS. The tissues were transferred to a sterile centrifuge tube containing a digestion solution composed of DMEM/F12 medium (Gibco, Grand Island, NY, United States) supplemented with 10% fetal bovine serum (FBS, Thermo Fisher Scientific Inc.), 2 mg/mL type II collagenase (Solarbio), 0.5 mg/mL type IV collagenase (Solarbio), and 100 IU/mL penicillin and streptomycin. The mixture was digested at 37 °C for about 60 min. After digestion, the suspension was filtered through a 70 μm cell strainer (Biosharp, Anhui, China), and the filtrate was collected in a new centrifuge tube. The cells were centrifuged at 200 × g for 5 min at room temperature, and the cells were collected, resuspended in PBS, and rinsed three times. Complete medium (DMEM/F12 medium containing 10% FBS and 100 IU/mL penicillin and streptomycin) was then added, and the cells were incubated at 37 °C in a 5% CO_2_ humidified incubator (33). Five Percoll density gradient layers (Sigma, St. Louis, MO, United States) with concentrations of 20, 30, 35, 40, and 60% were prepared. Then, 2.5 mL of cell suspension was gently layered on top and centrifuged at 200 × g for 30 min at 25 °C. Cells located between the 35 and 40% Percoll layers were collected and washed three times with DMEM/F12 medium. Each wash was performed by centrifugation at 200 × g for 5 min at 25 °C. Cell viability was determined by trypan blue exclusion, and only samples with > 85% viability were used. The cells were then resuspended in DMEM/F12 medium containing 10% FBS and 100 IU/mL penicillin–streptomycin, inoculated into 25 cm^2^ culture flasks (Corning, New York, USA), and incubated at 37 °C in a 5% CO₂ incubator (34, 35). When the cell density reached 80% (4–6 days), cells were detached with 0.25% trypsin–EDTA solution (Servicebio, Wuhan, China) and passaged for subsequent experiments.

### 2.2 Lentiviral infection screening and establishment of immortalized cell lines

The pLV–EF1α–Large T–antigen–PuroT plasmid (Addgene, Cambridge, United States) and its co-packaging plasmids pGag/Pol, pRev, and pVSV-G were kept in the laboratory. When the density of 293 T cells reached approximately 70%, plasmids pLV–EF1α–Large T antigen–PuroT (1.5 μg), pGag/Pol (1.5 μg), pRev (1.5 μg), and pVSV-G (0.5 μg) were combined with 7.5 μL of Lipofectamine 3,000 and 5 μL of P3000 reagent (Thermo Fisher Scientific, Shanghai, China) and transfected into the cells at 37 °C for 6 h. Cell supernatants containing viral particles were collected after 48 h, and cellular debris was removed by filtration through 0.45 μm filters. When the density of primary luteal cells reached approximately 70%, viral solution with multiplicity of infection (MOI) = 50 containing 6 μg/mL polybrene (Sigma-Aldrich, St. Louis, MO) was added. MOI = 50 was determined by preliminary titration experiments to achieve optimal infection efficiency. The cell culture medium was replaced with fresh medium 16 h after infection. Then, 5 μg/mL puromycin (Puro, Sigma-Aldrich, St. Louis, MO) was added for selection for 48 h. Puro selection was repeated twice, with medium replaced every 48 h, using the death of all cells in the control wells as the reference standard. After selection, cells were maintained in medium containing 2.5 μg/mL Puro to sustain selection pressure. The specific experimental methods and results for MOI, viral titer, and Puro can be found in the attachment. When the cell density reached 90%, Puro was removed, and the cells were continuously passaged up to passage 50 to obtain immortalized bovine luteal cell lines (18).

### 2.3 3β-HSD live-cell staining

PBLC and P30, P50 SV40T-IBLC cells were inoculated into 35 mm culture dishes. When the cell density reached 80%, the culture medium was discarded, and the cells were washed three times with PBS. Then, 1 mL of 3β-hydroxysteroid dehydrogenase (3β-HSD) staining solution was added. The staining solution was prepared by mixing 150 μL of 1.0 mg/mL nitroblue tetrazolium chloride (Thermo Fisher Scientific, Shanghai, China), 120 μL of 3.0 mg/mL NAD (Thermo Fisher Scientific, Shanghai, China), 50 μL of 10 mmol dehydroepiandrosterone (Aladdin, Shanghai, China), 100 μL of 1.6 mg/mL nicotinamide, and 580 μL of 0.01 mol/L PBS buffer (pH 7.4) to make a total volume of 1 mL. The cells were incubated with the staining solution in an incubator. After 4 h, the staining solution was discarded, and the cells were washed twice with PBS. Then, 1 mL of 4% paraformaldehyde (Biosharp, Anhui, China) was added to fix the cells for 20 min. Finally, the cells were washed twice with PBS and photographed under an inverted microscope (27).

### 2.4 Oil Red O staining

BLCs were inoculated in 35 mm culture dishes. When the cell density reached 80%, the culture medium was discarded. The cells were washed with PBS three times, fixed with 4% polyformaldehyde for 20 min, and washed with PBS three times. The cells were stained according to the instructions of the Oil Red O Staining Kit (Solarbio, Beijing, China) and were photographed under an inverted microscope.

### 2.5 Immunofluorescence identification and analysis

PBLC and P50 SV40T-IBLC were inoculated in special petri dishes for laser confocal microscopy (NEST, Wuxi, China) at approximately 80% confluency. The cells were fixed with 4% polyformaldehyde fixative for 15 min at room temperature, then incubated with PBS containing 0.3% Triton X-100 for 10 min at room temperature. Cells were blocked with 5% goat serum (Solarbio, Beijing, China) for 1 h. Subsequently, cells were incubated overnight at 4 °C with anti-oxytocin antibody, anti-synaptophysin antibody and anti-SV40 T antigen antibody (Abcam, Cambridge, MA, USA). After washing three times with PBS, cells were incubated with fluorescein isothiocyanate (FITC)-conjugated secondary antibodies for 1 h at room temperature. Protein staining was performed using FITC-coupled goat anti-rabbit IgG and Cy3-coupled goat anti-mouse IgG fluorescent secondary antibodies (Solarbio, Beijing, China) for 1 h. Cells were then incubated with BODIPY488 (Thermo Fisher Scientific, Shanghai, China) at room temperature for 30 min. Each step was washed three times with PBS. Finally, 4′,6-diamidino-2-phenylindole (DAPI) staining (Solarbio, Beijing, China) was performed for 5 min, and the results were observed using a laser confocal microscope (Nikon, Japan).

### 2.6 Cell growth curve and cell viability test

PBLC and P50 SV40T-IBLC were seeded in 96-well plates at a density of 1 × 10^3 cells per well. Each group included five replicate wells, and two independent replicate groups were prepared. After 24 h of pre-culture, cell viability was assessed using the Cell Counting Kit-8 (CCK-8) assay kit (AbMole, Shanghai, China) following the manufacturer’s instructions (36). The cells were incubated with the CCK-8 reagent for 2 h at 37 °C, after which absorbance at 450 nm was measured using a BioTek Synergy H1 microplate reader (United States). This measurement was repeated daily to monitor cell viability continuously over 8 days. Cell growth curves were plotted with incubation time on the horizontal axis and average optical density (OD) values on the vertical axis.

### 2.7 Effect of different concentrations of serum on cell viability

To investigate the effect of different concentrations of FBS on cell viability, P50 SV40T-IBLC cells were inoculated in 96-well plates at a density of 1 × 10^3 cells/well, with five replicate wells in each group and two biological replicates. After 24 h of pre-culture, the culture medium was replaced with DMEM/F12 basal medium. Following 6 h of serum starvation, the medium was replaced with complete medium containing different concentrations of FBS (0, 5, 10, 15, 20%). Cell viability was determined after 24 h of culture using the CCK-8 (AbMole, Shanghai, China) according to the manufacturer’s instructions. Specific methods for cell isolation and culture can be found in the articles published by the author (doi: 10.11843/j.issn.0366-6964.2024.05.039).

### 2.8 RNA extraction and quantitative reverse transcription

Quantitative reverse transcription PCR (qRT-PCR) was used to analyze gene expression. The expression of specific genes related to luteal function was detected in PBLC and P50 SV40T-IBLC. Total RNA was extracted from the cells using the Total RNA Extraction Kit (Vazyme Biotechnology, Shanghai, China). Reverse transcription was performed using the HiScript^®^ II First Strand cDNA Synthesis Super Mix for qPCR Kit (Vazyme Biotechnology, Shanghai, China). At least 1 μg of total RNA was used in a 20 μL reaction volume. ChamQ SYBR qPCR Master Mix kit (Vazyme Biotechnology, Shanghai, China) was used to pre-mix with cDNA according to the manufacturer’s instructions and then subjected to qRT-PCR analysis in a qRT-PCR system (Roche, Switzerland). The qRT-PCR conditions consisted of an initial denaturation at 95 °C for 2 min, followed by 40 cycles of 95 °C for 10 s and 60 °C for 30 s. GAPDH was used as an internal reference gene. The PCR primers used for quantitative analysis are listed in Table 1. The 2^ −ΔΔCt method was used to determine fold changes in mRNA expression (36).

**Table 1:** Primers used for real-time PCR.

Gene name	Sequence (5′ to 3′)	Product length	NCBI accession no.
*STAR*	F: GACGTTTAAGCTGTGTGCCG R: TTGGTCGCTGTAGAGAGGGT	202	NM_174189.3
*HSD3B*	F: CAGGAAATCCGGGTGCTAGA R: AACAGCAGCTGGGTACCTTTC	239	NM_174343.3
*CYP11A1*	F: ACCTGATTCCTGCCAAGACAC R: AGAATGTGGATGAGGAAGAGG	197	XM_010809903.3
*PTGFR*	F: GCACAGACAAGGCAGGTCTC R: CTGGCCATTGTCACCAGAAAA	105	XM_024984696.1
*PTGS2*	F: AGGGCTGGCAGGGTCG R: AGCCATTTCCTTCTCTCCTGTAAG	177	NM_174445.2
*PTGER*	F: TTCAGTGTCATCGTCAACCTCATC R: ATATATGCAAAAATCGTGAAAGGCA	194	NM_174588.2
*FGG*	F: AATGGAGTTTATTATCAAGGTGGCA R: GAACAGCTCAGGGCAAAATGA	250	NM_173911.2
*OXTR*	F: AGGAAGCCTCACCTTTCATCATC R: AGCGCTGCACAAGTTCTTGG	114	NM_174134.2
*LIF*	F: TCTCTGTCTTACAACACAGGCTCC R: TTTCCAGTGGAGAACCAGCAG	175	NM_173931.1
*TLR-4*	F: CAGGAAATCCGGGTGCTAGA R: CTGGATGATATTGGCGGCGATGG	124	DQ839567.1
*IL-6*	F: TCCAGAACGAGTATGAGG R: AGAATGTGGATGAGGAAGAGG	263	AC_000161.1
*ISG15*	F: GGTGAGGAACGACAAGGGTC R: CAGAATTGGTCCGCTTGCAC	176	NM_174366.1
*EGF*	F: GATCCTGCGTCCTTGGAACA R: ACAAAGGGTGAGCTGATGGG	208	XM_002688103.3
*VEGF*	F: ACATCACCATGCAGATTATGCG R: ACAGGGATTTTCTTGCCTTGC	128	NM_174216
*GAPDH*	F: CTACATGGTCTACATGTTCCAG R: CCTTCTCCATGGTAGTGAAGA	200	NM_001034034.2

### 2.9 Western blot

Western blot was performed as previously described (37). The membranes were incubated with the following primary antibodies: rabbit monoclonal antibody StAR (1:1000 in 5% BSA-PBST, Abcam, Cambridge, MA, United States); rabbit monoclonal antibody 3β-HSD (1:1000 in 5% BSA-PBST, Santa Cruz Biotechnology, Inc., CA, United States); murine monoclonal antibody SV40 T antigen (1:5000 in 5% BSA-PBST, Abcam, Shanghai, China); and murine monoclonal antibody PCNA (1:500 in 5% BSA-PBST, Proteintech, Wuhan, China). After washing, membranes were incubated with HRP-conjugated anti-rabbit, anti-goat, or anti-mouse secondary antibodies (1:10,000 in 1% BSA-PBS, Pierce, Rockford, IL, United States). Detection was performed using the Western Lighting ECL detection system (Pierce, Rockford, IL, United States). Signals were quantified using ImageJ software.

### 2.10 Progesterone assay

PBLC and SV40T-IBLC were seeded at 1×10^6 cells per well in 24-well plates. The culture medium was changed when the cell confluency reached about 60%. The supernatant was collected every 3 h. Three replicate wells were set up for each time point. The collected supernatants were centrifuged at 3000 rpm for 10 min and then filtered through a 0.45 μm filter membrane. The concentration of P_4_ in the culture supernatants was measured at different time points using an ELISA kit (Shanghai Enzyme-linked Biotechnology Co., Ltd., Shanghai, China) according to the manufacturer’s instructions. The minimum detection sensitivity was 0.03 ng/mL.

### 2.11 Karyotyping

Standard G-banding chromosome karyotyping was performed on PBLC and the P50 SV40T-IBLC. Metaphase chromosomes were visualized and analyzed using the ASI Chromosome Analysis System, developed in Israel.

### 2.12 Cell cycle detection by flow cytometry

PBLC and SV40T-IBLC were collected and centrifuged to remove the supernatant. The cells were then washed once with 0.01 mol/L PBS, and the supernatant was thoroughly removed. According to the Cell Cycle Assay Kit (MULTISCIENCES, Hangzhou, China), 1 mL of DNA staining solution and 10 μL of propidium iodide (PI) were added slowly. After shaking and mixing, the samples were incubated in the dark for 30 min. The staining solution was then filtered through a 45 μm cell strainer to remove large cell clusters. The filtrate was collected and analyzed by flow cytometry using a 488 nm excitation wavelength. Data were analyzed and plotted using FlowJo 10.8.1 software.

### 2.13 Soft agar assay and tumorigenic assay

The soft agar colony formation assay has been used to monitor immortalized luteal cells’ neoplastic transformation and anchorage-independent growth. The procedure on immortalized luteal cells was performed as previously described by Li et al. (19) for 2 weeks. As a positive control in this assay, HeLa cells were used. Colonies were measured and photographed with a phase-contrast microscope (Olympus, Beijing, China). Rate was calculated as (number of colonies/seeded cells × 100%).

We performed the experiments in strict accordance with the recommendations in the NIH Guide for the Care and Use of Laboratory Animals. This protocol was approved by the Ethics Committee for Animal Experimentation of Northwest A&F University (license no.: XN2023-0720). To analyze the potential tumorigenicity of immortalized luteal cells, we injected 1×10^6 SV40T-IBLC cells subcutaneously into the right posterior axillary region of ten 6-week-old female nude BALB/c mice. As a positive control, we injected the same number of MCF-7 cells into the right posterior axillary region of another ten 6-week-old female nude BALB/c mice. The mice were examined weekly for tumor formation over a period of one month. Tissues were sectioned and stained with H&E for pathological confirmation.

### 2.14 Modulation of bovine endometrial epithelial cells (BEEC) tolerance by SV40T-IBLC

BEEC were seeded into 6-well cell culture plates at a concentration of 1×10^5 cells/mL, and SV40T-IBLC were seeded into transwell chambers, then cultured separately for 24 h. BEEC and SV40T-IBLC were Co-cultured in 0.4 μm transwell inserts at a 1:2 ratio. The two types of cells were Co-cultured for 48 h and 72 h. Cell viability was assessed using the CCK-8 assay. Lipid droplet density in the cells was determined by Oil Red O staining, and the apoptosis rate was measured by flow cytometry assay. Total cellular RNA was extracted, and the mRNA expression of genes related to endometrial tolerance, such as TLR-4, IL-6, LIF, EGF, VEGF, and ISG15, was detected.

### 2.15 Data analysis

The experimental data were analyzed using IBM SPSS 26 and GraphPad 9.5 software. The data were presented as mean ± standard deviation (mean ± SD). The experimental data were found to follow a normal distribution, and variance homogeneity tests were conducted. The means of two groups were compared using an independent samples Student’s t-test. Pairwise comparisons between groups were performed using the Bonferroni test. A significance level of *p* < 0.05 was considered statistically significant.

## 3 Results

### 3.1 Isolation and purification of PBLCs and *in vitro* culture

**Figure 1:**
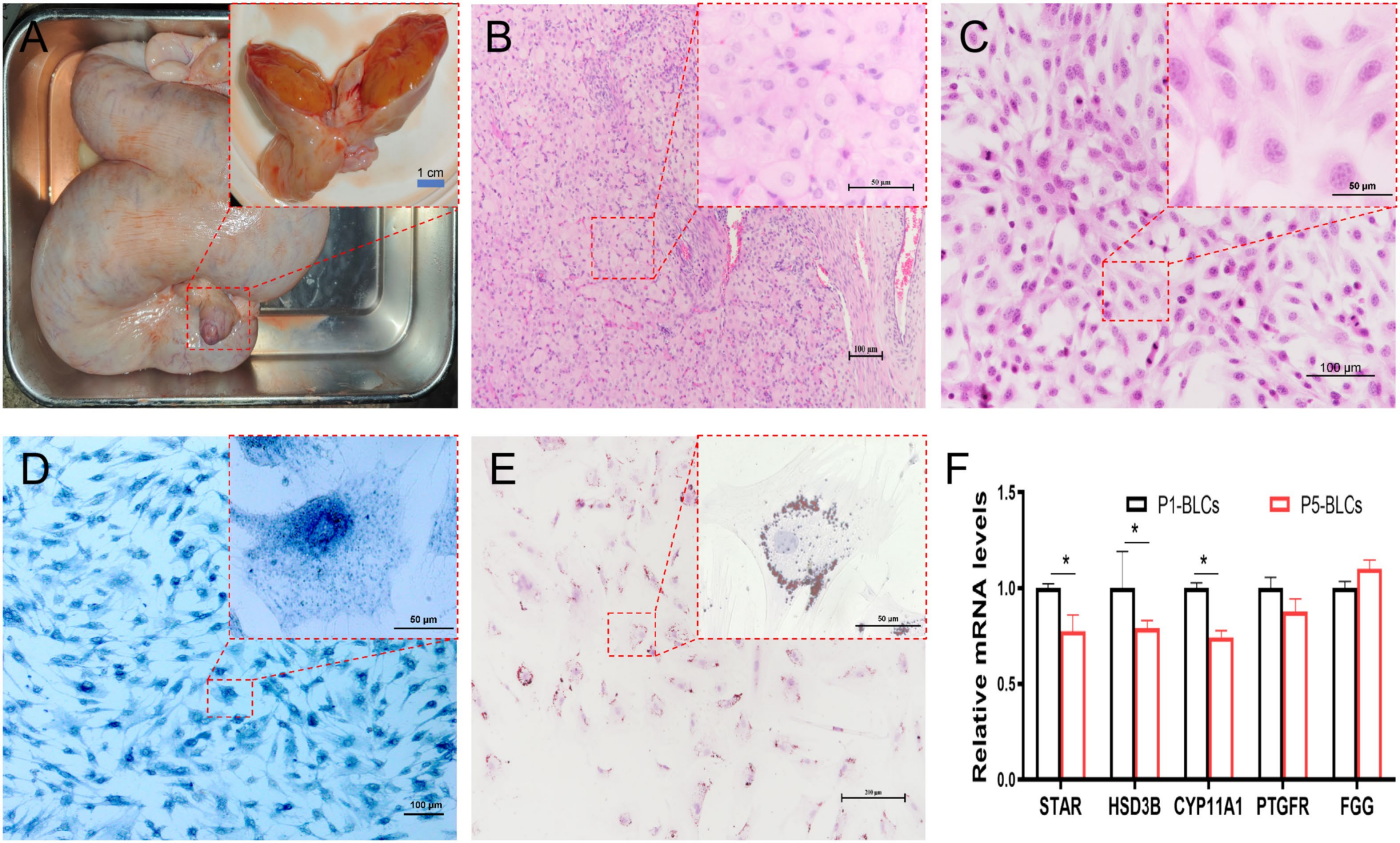
Isolation and characterization of primary luteal cells. **(A)** Anatomical Morphology of the Bovine Corpus Luteum During Pregnancy (*n* = 3 per group); **(B)** HE-stained histology of luteal tissue from pregnant dairy cows (*n* = 3 per group); **(C)** morphological Characteristics of BLC Cultured *in vitro*; **(D)** staining results for BLC 3β-HSD *in vitro* cultured cells; **(E)** distribution Characteristics of Oil Red O-Stained Lipid Droplets in BLC; **(F)** expression characteristics of marker genes in primary BLCs cultured *in vitro* (P1 and P5) (*n* = 3 per group). Data represent the mean ± SEM (experiments were repeated 3 times independently with triplicate technical replicates). * *p* < 0.05, ** *p* < 0.01.

The CL during pregnancy has a rich blood vessel distribution, and tissue sections show a large number of BLCs (Figures 1A,B). After enzymatic digestion and purification using Percoll centrifugation, luteal cells cultured *in vitro* maintained their primary biological characteristics and expressed a series of genes specific for luteal cells (Figures 1C,F). These cells exhibit an irregular polygonal morphology, with large nuclei and abundant cytoplasm, typical of epithelial-like cells; a large number of lipid droplets were diffusely distributed in the cytoplasm (Figure 1E). Both 3β-HSD staining and Oil Red O staining showed that highly pure primary luteal cells were successfully isolated and purified using collagenase digestion combined with Percoll discontinuous density gradient centrifugation (Figure 1D).

### 3.2 Immortalization of PBLC

**Figure 2:**
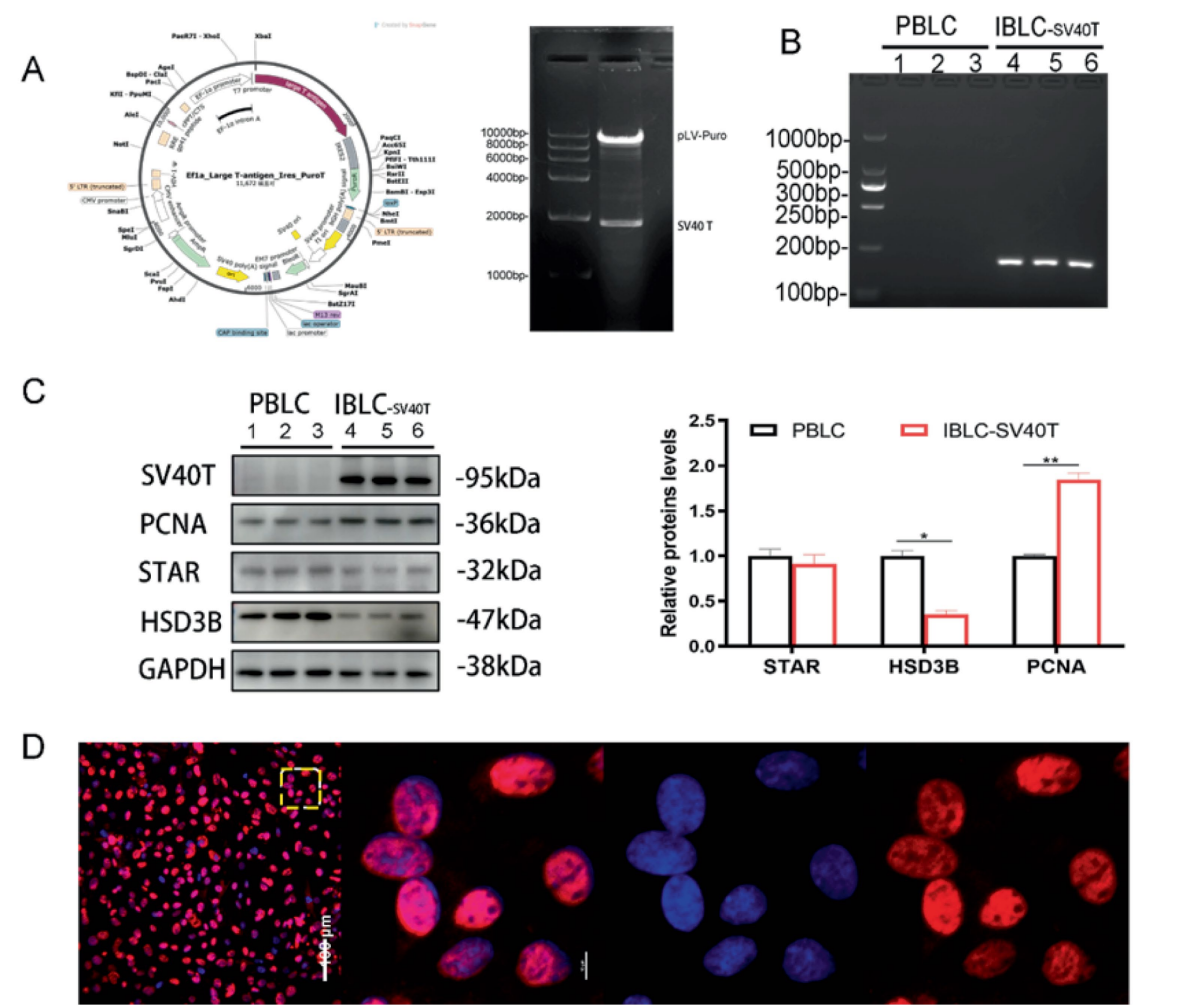
Lentiviral infection to construct immortalized luteal cell lines. **(A)** Plasmid map and restriction enzyme gel electrophoresis results of PLv-EF1α-SV40T-Puro; **(B)** qPCR results of the SV40 T antigen gene and gel electrophoresis and quantitative analysis of its products; **(C)** western blotting detection of SV40 T antigen expression and quantitative analysis results; **(D)** immunofluorescence detection of SV40 T antigen localization in BLC. Data represent the mean ± SEM (experiments were repeated 3 times independently with triplicate technical replicates). *p <0.05, **p < 0.01.

The plasmid sequence was identified by enzymatic cleavage and confirmed to match the target gene sequence, ensuring its suitability for subsequent experiments (Figure 2A). The pseudovirus was packaged in 293 T cells. Using the minimum concentration and time at which all control group cells die as the Puro screening conditions, Puro selection identified 5 μg/mL, with the screening lasting 48 h, resulting in a Puro-resistant positive cell population after screening (the specific methods and standards can be found in the attachment). After puromycin screening, positive cell populations were subjected to limiting dilution in 96-well plates at a density of 0.5 cells per well to isolate single-cell clones. Single clones with uniform epithelial-like morphology, stable SV40 T antigen expression (verified by qPCR and Western blot), and consistent progesterone secretion capacity were selected for serial passage. The final cell line used in all subsequent experiments was derived from a single monoclonal cell with stable proliferation and functional characteristics consistent with primary bovine luteal cells up to passage 50 (Figures 2B–D).

### 3.3 Biological properties of immortalized luteal cell lines

**Figure 3:**
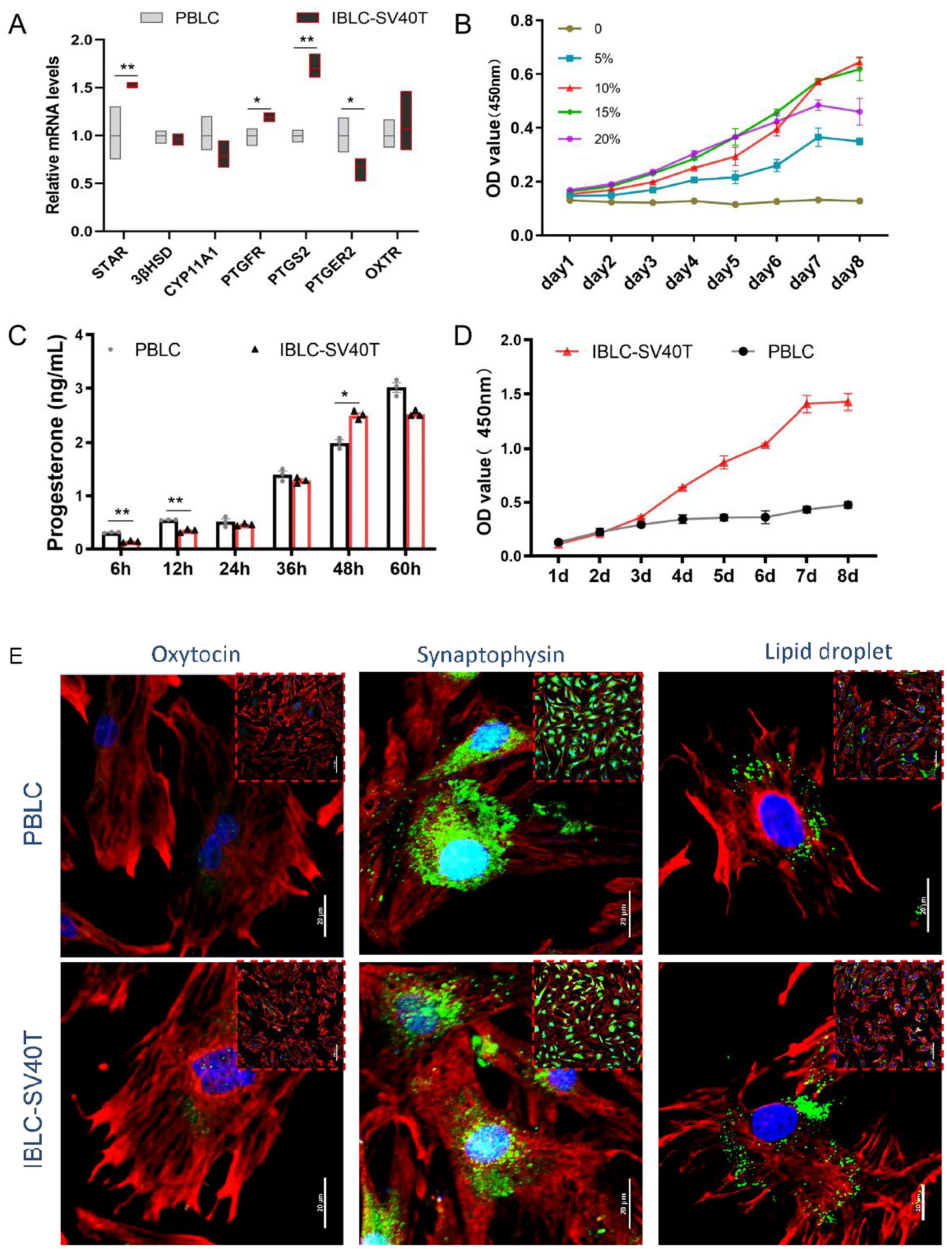
Biological properties of immortalized luteal cell lines. **(A)** Results of qPCR detection of key genes involved in P_4_ synthesis in PBLC and SV40T-IBLC (*n* = 3 per group); **(B)** Serum dependence of SV40T-IBLC (*n* = 6 per group); **(C)** ELISA detection of P_4_ concentration in the culture supernatant of PBLC and SV40T-IBLC cells (*n* = 3 per group); **(D)** Growth curves of SV40T-IBLC and PBLC (*n* = 5 per group); **(E)** Distribution characteristics of oxytocin, synaptophysin, and lipid droplets in BLC (*n* = 3 per group). Data represent the mean ± SEM (experiments were repeated 3 times independently with triplicate technical replicates). * *p* < 0.05, ** *p* < 0.01.

To evaluate whether continuous *in vitro* passaging after immortalization would alter the main biological properties of BLC, PBLC were used as a control to assess their main biological functions from multiple perspectives. The results showed that SV40T-IBLC had similar biological properties to the primary cells. At the gene level, SV40T-IBLC expressed a variety of BLC-specific marker genes, including PTGFR, CYP11A1, FGG, and OXTR (Figure 3A). At the protein level, they expressed key steroidogenic enzymes such as STAR and 3β-HSD (Figure 3C), as well as luteinizing luteal cell-specific marker proteins OT and SYP (Figure 3E). Functionally, SV40T-IBLC displayed lipid droplet distribution in the cytoplasm similar to that of progenitors and maintained a time-dependent capacity for P_4_ secretion (Figures 3C,E). Meanwhile, PBLC were limited as stable cell models because of their weak proliferative capacity and inability to undergo extended passaging. The decline in PBLC OD values after day 5 reflects their limited proliferative capacity and senescence. In contrast, the *in vitro* culture of SV40T-IBLC still required serum supplementation and FBS dependence but exhibited significantly enhanced proliferative ability and extended lifespan due to SV40 T antigen expression, thereby overcoming the generational barrier of PBLCs (Figures 3B,D).

### 3.4 Transformation characteristics of immortalized luteal cells

**Figure 4:**
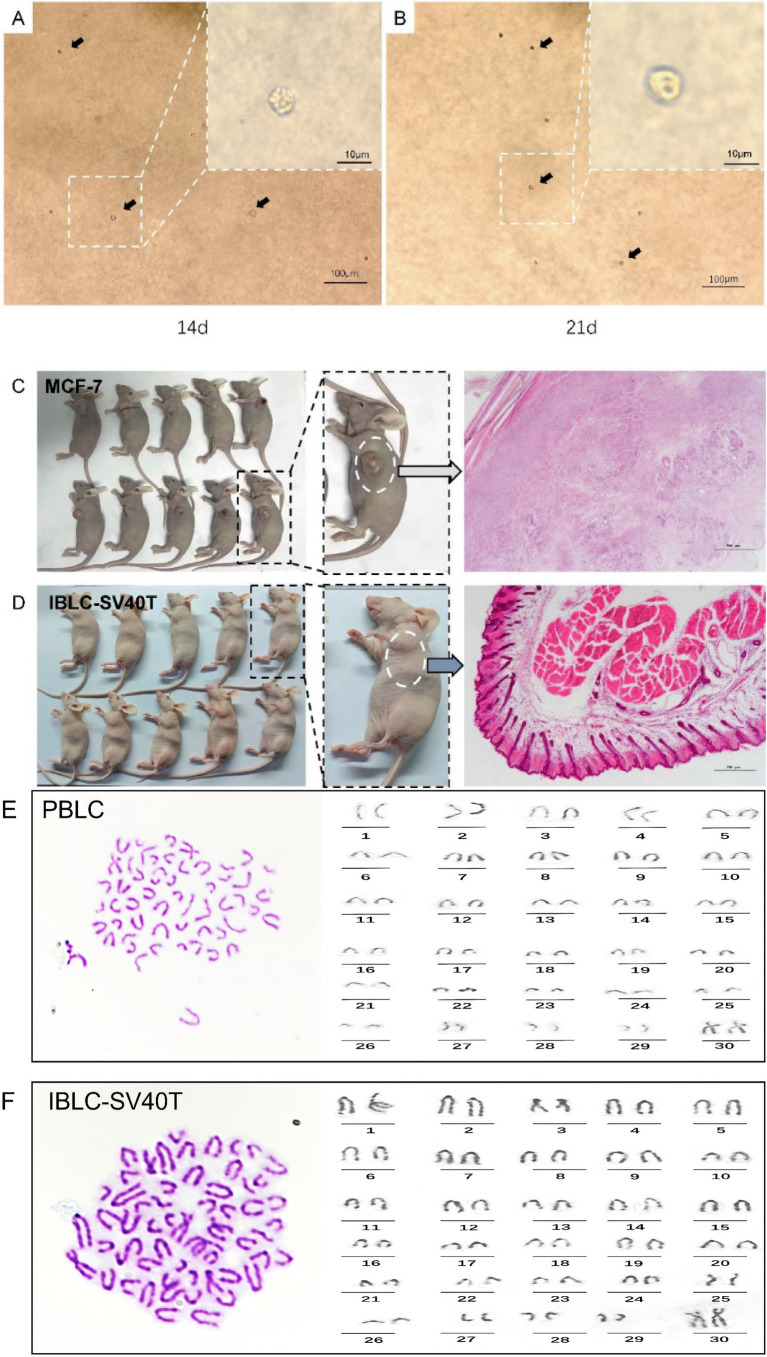
Transformation properties of immortalized luteal cells. **(A)** Morphology of SV40T-IBLC after 14 days of colonization in soft agar (*n* = 3 per group); **(B)** morphology of SV40T-IBLC after 21 days of colonization in soft agar (*n* = 3 per group); **(C)** tumor masses formed by MCF-7 in the axillary subcutaneous tissue of nude mice and HE staining results of tissue sections (*n* = 10 per group); **(D)** tumor masses formed by SV40T-IBLC in the axillary subcutaneous tissue of nude mice and HE staining results of tissue sections (*n* = 10 per group); **(E)** PBLC metaphase plates and karyotypes; **(F)** SV40T-IBLC metaphase plates and karyotypes (*n* = 3 per group). Data represent the mean ± SEM (experiments were repeated 3 times independently with triplicate technical replicates). * *p* < 0.05, ** *p* < 0.01.

To assess whether immortalized luteal cells had undergone malignant transformation, chromosome karyotype analysis was performed on the P50 generation cells. The results showed that the chromosome number was diploid, consisting of 29 pairs of autosomes and one pair of XX sex chromosomes, with no detectable chromosomal abnormality (Figures 4E,F). SV40T-IBLC were further tested for anchorage-independent growth by a soft agar assay. After 21 d, the BLC in the agar remained singly distributed, and showed transparent round refractive bodies under the inverted microscope, maintaining anchorage-dependent growth characteristics (Figures 4A,B). In addition, human breast cancer MCF-7 cells and SV40T-IBLC were inoculated subcutaneously into the post-axillary region of the right forelimb of 6-week-old nude mice. After 30 d, tumors formed at the injection sites of the MCF-7 group, whereas no tumors developed in the SV40T-IBLC group. Histological observations indicated that in nude mice inoculated with MCF-7 cells, examination of tissue pathology sections revealed disordered cell arrangement, loss of polarity, and numerous mitotic figures (Figure 4C). In contrast, the subcutaneous tissue of mice inoculated with the SV40T-BLC cell line showed clear structure without pathological changes (Figure 4D). The injection sites displayed normal histological architecture, indicating that SV40T-IBLC did not exhibit tumorigenic potential in the *in vitro* anchorage-independent growth assay and *in vivo* nude mouse tumorigenicity assay (Figures 4C,D).

### 3.5 Immortalized luteal cell line modulates endometrial cell tolerance

**Figure 5:**
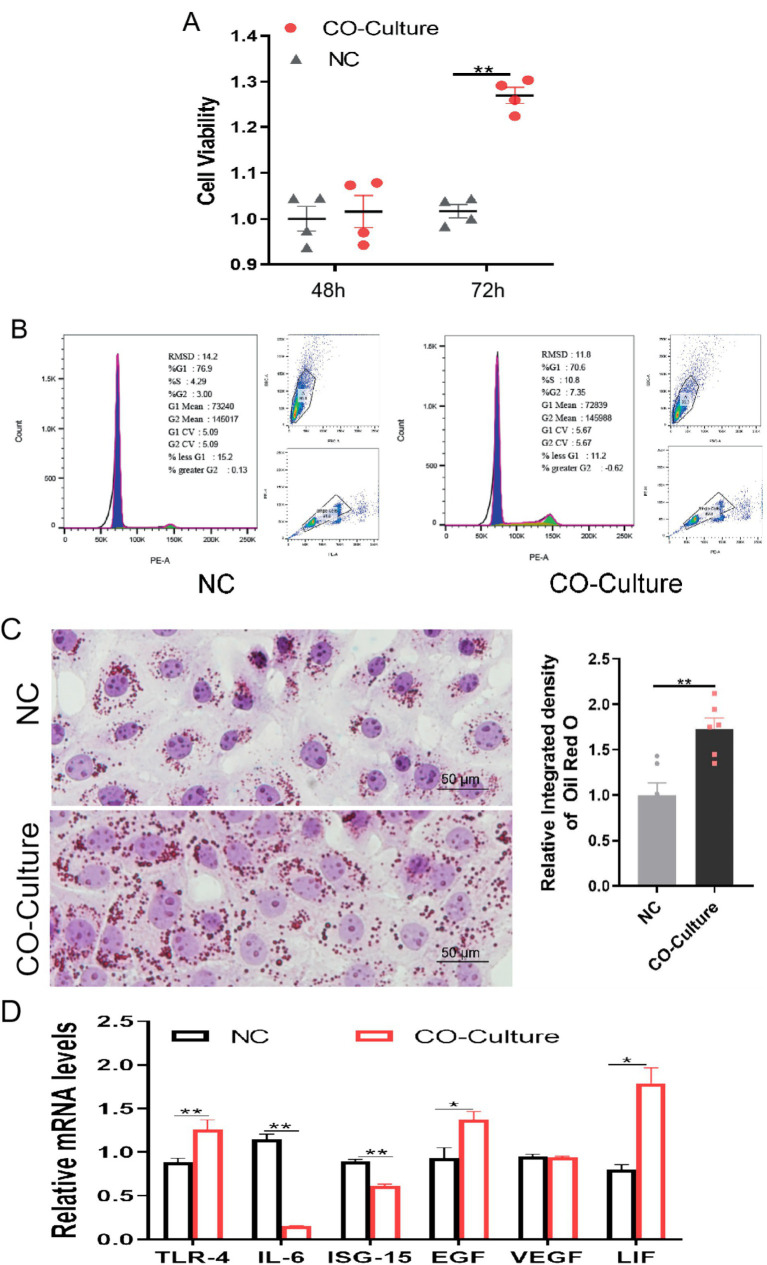
Immortalized luteal cell line modulates endometrial cell tolerance. **(A)** CCK-8 detection of co-cultured BEEC cell vitality results (*n* = 4 per group); **(B)** Flow cytometry detection of co-cultured BEEC cell cycle results; **(C)** LD distribution oil red O staining results and quantitative analysis diagram of BEEC (*n* = 3 per group); **(D)** qPCR detection of co-cultured BEEC tolerance-related gene expression characteristics (*n* = 3 per group). Data represent the mean ± SEM (experiments were repeated 3 times independently with triplicate technical replicates). * *p* < 0.05, ** *p* < 0.01.

Luteal cells secrete P_4_, estrogen and various cytokines that regulate the function and proliferation of endometrial epithelial cells. In this study, SV40T-IBLC were Co-cultured with BEEC to evaluate their regulatory effects. The results showed that luteal cells significantly promoted the functional activity of BEEC, as evidenced by a marked increase in BEEC viability after 72 h of Co-culture (*p* < 0.01) and an increased abundance of lipid droplets in the Co-cultured group compared with endometrial cells cultured alone (*p* < 0.01). But after Co-culturing for 48 h, there was no significant change in cell viability (Figure 5A). Lipid droplet accumulation, a key indicator of steroidogenic activity, provides essential substrates and structural platforms for steroid hormone synthesis (Figure 5C). Moreover, SV40T-IBLC modulated the expression of genes associated with endometrial immune tolerance. Although the expression of IL-6 and ISG15 remained low (*p*>0.05), the expression of EGF and LIF was elevated (*p* > 0.05), indicating that BLCs could enhance endometrial immune tolerance and support the viability of BEEC (Figure 5D). Importantly, co-culture had no significant effect on BEEC apoptosis rates (Figure 5B).

## 4 Discussion

### 4.1 Isolation and culture of BLC

There are many types of cells in luteal tissue, and suitable cell isolation and purification methods can provide primary cells in good condition, in larger numbers, and from a homogeneous source for subsequent studies (38). The isolation methods of luteal cells mainly include two types of method, enzyme digestion and tissue block, with digestion of luteal tissue with type II collagenase to obtain cells most often (19, 39), but in this study, it was found that the use of type II collagenase alone was not effective in digesting bovine luteal tissue, which may be related to the interspersed luteal tissues with a large number of connective tissues, and the combination of type II and type IV collagenase can shorten the digesting time while obtaining a large number of well-conditioned luteal cells (23, 40). The combined use of type II and type IV collagenase can shorten the digestion time and at the same time obtain a large number of luteal cells in good condition. The methods that have been reported for the isolation and purification of luteal cells include differential digestion, immunomagnetic bead separation, and centrifugal elutriation (26), whereas Percoll discontinuous density gradient centrifugation has been commonly used for the purification of animal luteal cells due to its convenience, efficiency, and inexpensiveness (15, 40). In this experiment, by setting up Percoll sorting solution with different density gradients, the erythrocytes, fibroblasts, vascular endothelial cells, and many other cells in the separated cell suspension can be effectively removed by simple low-speed centrifugation, and the luteal cells with 95% purification rate can be obtained from 35–40% of the cell suspension.

### 4.2 Lentivirus for the establishment of immortalized luteal cell lines

In this study, the SV40 T antigen gene was transduced into BLCs using a lentiviral vector, integrating the SV40 T antigen gene into the chromosomal genome of BLCs, enabling BLCs to stably express the SV40 T antigen gene without generational loss, thereby successfully establishing a stably transfected immortalized bovine luteal cell line (SV40T-IBLC).

Among the various methods for establishing immortalized cell lines through exogenous gene transduction, the method of immortalization by transduction of hTERT is considered a safe and efficient method for immortalizing cell lines due to its ability to maintain the normal karyotype and physiological characteristics of cells (36, 41). However, in this study, SV40 T antigen was prioritized for immortalization of BLC because it can efficiently overcome replicative senescence in primary cells by simultaneously inhibiting two key senescence pathways, p53 and Rb, and has a high transformation success rate and mature technical pathway in a variety of mammalian cells (24, 36, 41, 42). SV40 T antigen binds to and inactivates tumor suppressor proteins p53 and pRb family members, thereby releasing cell cycle arrest and senescence signals, promoting sustained cell proliferation (42). This mechanism belongs to the classical strategy of “bypassing/inhibiting the p53-p16-Rb stress response,” which is more likely to obtain a stable immortalized phenotype in some cell types than activated telomerase (TERT) alone (43). In addition, stable expression of SV40 T antigen usually does not completely inhibit differentiation potential, and removal of T antigen can also improve terminal differentiation (44). This characteristic makes it suitable for research models that require the restoration of physiological functions after amplification (43).

SV40 T antigen and TERT are not mutually replaceable, but are a strategy choice, and TERT prolongs lifespan by maintaining telomere length, but its use alone is not as stable as the strategy of “inhibiting p53/p16/Rb” in some cells (41). The advantage of SV40 T antigen is that it directly unlocks cell cycle checkpoints, so it is often prioritized when rapid access to stable proliferative cell lines is required. TERT, on the other hand, is more suitable for use in combination with checkpoint suppression strategies to improve success rates and genetic stability (43, 44). In most terminally differentiated cells, telomerase is extensively inhibited, and telomerase activity is undetectable within cells (20). The method of cell immortality by transducing hTERT is only suitable for cells that proliferate in a telomerase-dependent manner. For such cells, TERT transduction alone cannot achieve immortalization. BLCs are typical terminally differentiated cells, and telomerase activity is undetectable in their cells (21). This study also tried to establish immortalized cell lines by transducing hTERT, but BLC cells entered the quiescent phase after transduction and could not be passed continuously. This limits subsequent comparative studies to establish immortalized BLC cell lines by transducing hTERT and SV40 T antigen genes. There may be two reasons for this result: On the one hand, BLC is a terminally differentiated cell with a highly specialized nucleus. Its method of maintaining telomeres relies on alternative lengthening of telomeres rather than reverse addition of telomerase (22). On the other hand, the efficiency of human hTERT and bovine bTERT in inducing bovine fibroblast immortalization, bTERT is more effective than hTERT in achieving immortalization of primary cells in large animals such as cattle and sheep (16). This suggests that there may be interspecies differences in inducing cell immortalization of heterologous TERT genes.

Cell transformation induced by the SV40 T antigen is a complex process, which may induce the biological characteristics of the cells to change as they enter into an immortalized state and induce transformation of the biological properties of the cells (37). In the present study, the results of soft agar cloning and nude mice tumor assay showed that the cell line retained its anchored growth characteristics and was unable to form tumors and no evidence of malignant transformation was observed in the performed assays, while the karyotypic sorting of its chromosomes verified that there were no chromosomal aberrations and confirmed that the cell line originated from bovine.

### 4.3 Biological phenotypes of immortalized luteal cells

Luteal cells are typical neuroendocrine cells with a typical epithelial cell morphology, huge size and abundant cytoplasm; they have a variety of specific phenotypes (45), and they are model cells for the study of multi-system interactions and the regulation of reproduction. But a recent study also confirms that only short-term luteal cell primary cultures or very early passaged luteal cells (P3) are able to display molecular features that resemble that of the corpus luteum *in vivo* and thus are suitable *in vitro* models (46), In this study, it was found that the P_4_ synthesis capacity of luteal cells in early culture increased in a time-dependent manner, but their P_4_ synthesis capacity gradually decreased in long-term culture, which may be related to the direct uptake of cholesterol for steroid synthesis by bovine luteal cells directly from the outside world. It has been well documented to support this inference that the steroid synthesizing ability of luteal cells can be enhanced in a dose-dependent manner by the additional addition of 22(R)-hydroxycholesterol to goat luteal cells cultured *in vitro* (47). Therefore, the SV40T-IBLC established in this study shows an irreplaceable species-specific advantage in reproductive medicine, drug metabolism, and disease mechanism research, providing an ideal model for the development of bovine-derived biological products and the study of pregnancy-related diseases.

The formation of lipid droplets is a hallmark of pathological conditions, such as cholesterol ester-containing foamy macrophages in atherosclerotic lesions and fatty liver disease due to hepatic injury (48), but in steroidogenic tissues, such as luteal tissues, the diffuse distribution of small lipid droplets, is a major feature of luteal cells. It has been found that luteal lipid droplets are a distinguishing feature of bovine luteal cells from other luteal constitutive cells, and there is a difference in the characteristics of lipid droplets in large and small luteal cells; large luteal cells contain many diffusely distributed small lipid droplets, whereas small luteal cells contain a small number of small lipid droplets with a large size, which is presumed to be related to the functional compartmentalization of luteal cells (49). In the present experiment, the identification of isolated luteal cells also revealed phenomena consistent with the above.

In this study, luteal cells obtained from isolation and purification were characterized by 3β-HSD live-cell staining and Oil Red O lipid droplet staining, and 3β-HSD was found to overlap with the location of lipid droplet distribution. And a recent study has found that lipid droplets are a new platform for steroid synthesis in bovine luteal cells (50). The key proteins in P_4_ biosynthesis include steroidogenic acute regulatory protein (STAR), cytochrome P450 family 11 subfamily A member 1 (CYP11A1) and 3 beta-hydroxysteroid dehydrogenase (3β-HSD) (51). In contrast, the abundant distribution of active CYP11A1 and 3β-HSD in the lipid droplets of luteal cells provides conditions for the catalytic synthesis of P_4_ from pregnenolone directly in the lipid droplets (52). P_4_ synthesized by luteal cells requires cholesterol as a substrate, and cholesterol needs to be transported from cytoplasmic lipid droplets to mitochondria. This process is jointly accomplished by StAR and hormone-sensitive lipase (HSL). LH promotes HSL phosphorylation and activation by activating the cAMP/PKA signaling pathway. HSL hydrolyzes cholesterol esters in lipid droplets, releasing free cholesterol (FC) to provide cholesterol substrate for mitochondria. FC needs to be transported from the outer mitochondrial membrane to the inner membrane via StAR, which is the rate-limiting step in steroid hormone synthesis. StAR is located on the outer mitochondrial membrane and can bind FC, transporting it to the inner membrane through a conformational change, providing raw materials for subsequent progesterone synthesis. This regulation ensures that luteal cells can efficiently synthesize progesterone when energy is sufficient and reduce consumption when energy is insufficient, maintaining hormonal balance during pregnancy. In addition to steroid hormone synthesis function, luteal cells can also specifically secrete the peptide hormone oxytocin, and express synaptophysin, a key marker protein of mature functional luteal cells (34, 53).

And some studies based on transcriptomics found that in luteal cells, genes such as OXTR, PTGER, PTGFR, and FGG showed an order of magnitude difference between LLC and SLC (35), and that the major luteal cell is the main cell for the maintenance and regulation of luteal function, and that SLCs can be directed to differentiate into the LLCs (54). An ideal immortalized cell line is one that is able to maintain similar biological properties to primary cells, thus mimicking the major biological properties of cells *in vivo* while reducing experimental errors (13, 37). In using PBLCs as a control, IBLC were still able to stably express luteal cell-specific genes and proteins, such as OT, SYP, PTGFR, etc., when cultured *in vitro* up to P50, and retained the similar morphology and biological characteristics of the primary generation.

### 4.4 Co-culture of SV40T-IBLC with BEEC

Studies have found that P_4_ can significantly regulate the expression of genes related to endometrial receptivity in BEECs, and similar results were observed in this study (55). Endometrial receptivity is regulated not only by P4 but also by multiple factors from LC in a coordinated manner (56). In Co-culture studies, it was found that Co-culture not only enhanced the expression of endometrial receptivity-related genes in BEECs but also significantly increased BEEC cell viability and proliferation, whereas BEECs treated with P_4_ alone showed no significant change in cell viability and proliferation. This suggests that BEECs in Co-culture are regulated not only by P4 secreted from BLC but also by other signaling molecules. In addition to synthesizing and secreting various hormones such as P4, E2, and OT, luteal cell can also produce exosomes, microRNAs, lncRNAs, cytokines, and other signaling molecules, which are involved in regulating endometrial receptivity (57). Under natural physiological conditions, the interaction between LC and BEEC involves not only changes in hormone levels but also the collaborative action of various signaling molecules (58, 59). In this study, by constructing a Co-culture system of SV40T-IBLC and BEEC, the endometrial environment under natural physiological conditions was simulated to a certain extent. This model can provide a cellular model for further study of the coordinated communication between the uterus and ovary during the luteal phase.

### 4.5 Research limitations

This study has the following limitations. First, the immortality strategy is singular, and parallel experiments with other immortalization strategies (such as hTERT-mediated telomerase activation) were not conducted, making it impossible to comprehensively compare the advantages and disadvantages of different methods and the differences in cell line characteristics. Second, long-term passage stability data are limited, with validation only up to P50 for proliferation and functional stability, lacking assessments of long-term passage stability beyond P50, so the risk of long-term genetic drift has not been completely excluded. Additionally, there is a lack of *in vivo* functional validation; the study focused on *in vitro* characterization and did not verify the *in vivo* function of this cell line through animal models, so its physiological relevance requires further *in vivo* evidence. These aspects need to be further improved in subsequent work.

## 5 Conclusion

In summary, this study establishes an immortalized bovine luteal cell line that proliferates stably *in vitro* and retains similar biological characteristics to the primary bovine luteal cells. The cell line has the same morphology as the primary bovine luteal cells and can stably secrete hormones such as P_4_ and oxytocin. It also regulates the immune tolerance of endometrial cells cultured *in vitro*. This model can be used to study the regulation of luteal function in dairy cows, explore new reproductive management strategies, investigate the mechanisms of nutritional regulation, and develop new genetic improvement methods, thereby providing a model of bovine luteal cells with a uniform genetic background and stable proliferation.

## Data Availability

The original contributions presented in the study are included in the article/Supplementary material, further inquiries can be directed to the corresponding authors.
