# Supplementary material for: Establishment and characterization of an immortalized bovine luteal cell line
Source: Front Vet Sci. 2026 May 18;13:1758622. doi: 10.3389/fvets.2026.1758622 (PMC13222825; doi:10.3389/fvets.2026.1758622)
Supplement: Supplementary file 1 [file Data_Sheet_1.docx]

1. Puromycin Optimal Concentration Screening

1.1 Method

(1) Primary luteal cells were adjusted to a specific cell density and seeded at 1×10^5 cells/mL in a 24-well cell culture plate, then incubated overnight;

(2) Preparation of selection medium: Dilute Puro with complete medium to a range of 0 ~ 10 μg/mL, setting one selection concentration for every 1 μg/mL increment;

(3) After overnight incubation, discard the medium and add the selection medium to incubate the cells;

(4) Observe the cell growth status and replace the selection medium every 2 days;

(5) Count the cell survival rate every 24 hours;

(6) The lowest selection concentration that kills all cells within 4 days of selection is used as the optimal selection concentration.

1.2 Results

Ten Puro selection concentrations of 1, 2, 3, 4, 5, 6, 7, 8, 9, and 10 μg/mL were set, and the culture medium was replaced every 2 days, constituting one selection cycle. After two cycles, cells in the 1–4 μg/mL selection groups survived, while cells in the 5–10 μg/mL selection groups all died. The lowest completely lethal concentration of 5 μg/mL was chosen as the optimal selection concentration for subsequent cell selection.

2 SV40T Antigen Immortalization Lentivirus Titer Determination

2.1 Method

(1) Culture HEK-293T cells to the logarithmic growth phase;

(2) After trypsinizing and resuspending the cells, adjust the cell density and seed 1 × 10^5 cells/mL into a 12-well culture plate. Incubate overnight at 37°C. For infection, the cells should reach a confluence of 20%–30%;

(3) After 24 hours, perform the transfection on the 12-well plate. Thaw the virus stock stored at -80°C in an ice bath and perform a serial dilution using DMEM without serum;

(4) Group the wells, gently mix each tube of diluted lentivirus, add 100 µL of virus suspension to each well, and incubate overnight;

(5) After 72 hours, observe the cell status. When the cells are healthy and the density exceeds 80%, collect the cells for subsequent qPCR.

(6) qPCR Detection

The specific procedures for total RNA extraction and reverse transcription experiments are described in Appendix B. Quantitative primers are designed in the WPRE sequence region of the vector, using GAPDH as an internal reference gene; qPCR is used to measure the WPRE copy number in recombinant lentivirus to determine the number of viral particles. The primer sequences are shown in Table 2-1.

Table 2-1 Primer Sequences

| Gene Name | Primer Sequence（5'-3'） | NCBI  Accession NO. |
| --- | --- | --- |
| WPRE | F: CGCTATGTGGATACGCTGCTTTA  R: CGGCGAAGATAGCGGCATTA | NM_173931.1 |
| GAPDH | F: CTACATGGTCTACATGTTCCAG  R: CCTTCTCCATGGTAGTGAAGA | NM_001034034.2 |

(7) Data Analysis: The copy number of the sample to be tested can be determined based on its fluorescence intensity, using this standard curve.

2.2 Results

Four standard dilution gradients were set, containing 1 × 10^-2 mL, 1 × 10^-3 mL, 1 × 10^-4 mL, and 1 × 10^-5 mL of standard lentivirus solution; qPCR detected CT values are shown in Table 2-2:

Table 2-2 Viral titers of standards

| Standard Product | GAPDH  (CT value) | WPRE  (CT value) | Relative titer | Titer  (= Relative titer × Dilution factor) |
| --- | --- | --- | --- | --- |
| 1 × 10^-2 mL | 16.10 | 13.12 | 1 × 10^9 | 1 × 10^9 |
|  | 16.40 | 13.16 |  |  |
| 1 × 10^-3 mL | 15.63 | 16.54 | 1 × 10^8 | 1 × 10^9 |
|  | 15.95 | 16.72 |  |  |
| 1 × 10^-4 mL | 15.46 | 19.56 | 1 × 10^7 | 1 × 10^9 |
|  | 15.49 | 19.95 |  |  |
| 1 × 10^-5 mL | 16.47 | 23.21 | 1 × 10^6 | 1 × 10^9 |
|  | 16.50 | 23.09 |  |  |
| Control | 16.46 | 33.11 | / | / |
|  | 16.95 | 32.78 |  |  |

Add 50 μL of viral stock solution and 5 μL of virus-treated 293T cell samples, then perform qPCR detection. The titer of the virus sample group is shown in Table 2-3:

Table 2-3 Plv-SV40T-Puro Lentivirus Titers

|  | GAPDH  (CT value) | | WPRE  (CT value) | | Titer |
| --- | --- | --- | --- | --- | --- |
| 50 μL | 14.33 | 14.45 | 16.12 | 16.35 | 1.31 × 10^8 |
| 5 μL | 14.33 | 14.10 | 19.58 | 19.77 | 1.01 × 10^8 |

In summary, the Plv-SV40T-Puro lentivirus was successfully packaged, with a titer of 1.16×10^8 TU/mL.

3 Determination of the Optimal Multiplicity of Infection (MOI) for BLC

3.1 Method

(1) Revive PBLCs and adjust the cell density. Seed cells into a 24-well plate at a density of 1×10^5 cells/mL. After 48 hours of culture, when the cell density reaches approximately 60%, proceed with subsequent experimental operations.

(2) Prepare 1.5 mL sterile EP tubes, adding 125 µL of serum-containing DMEM/F12 medium to each tube. Take the GFP-labeled virus from the -80 ℃ freezer and thaw it in an ice bath. Then, sequentially add 5, 10, and 20 µL of virus solution (virus titer 5×10^8 TU/mL), gently pipette to mix, and set aside.

(3) Discard the culture medium, add fresh medium at half volume, and drop the virus solution into the cell culture wells. Incubate in the cell culture incubator.

(4) After 4 hours, add medium to reach full culture volume and continue incubation at 37 ℃ for 24 hours.

(5) After 48 hours, perform preliminary fluorescence observation under a microscope.

(6) After 72 hours, for groups with approximately 80% infection efficiency and good cell growth, the corresponding infection conditions and MOI (MOI = virus titer × virus volume / cell number) can serve as a reference for subsequent infection experiments.

3.2 Results

Three lentiviral infection gradients were set with MOI of 25, 50, and 100. After 72 hours of viral infection, an inverted fluorescence microscope was used to observe and count the cell fluorescence expression and cell growth status. It was observed that after 72 hours, in the group treated with MOI = 50, the cell fluorescence distribution was approximately 80%; thus, the optimal multiplicity of infection (MOI) for PBLCs was determined to be 50 (Figure 3-2).

| 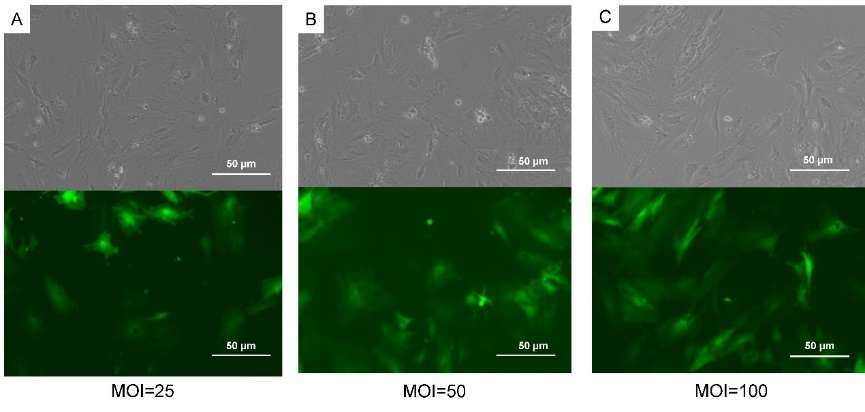 |
| --- |
| Figure 3-2 BLC optimal multiplicity of infection determination  A. Results of GFP virus infection with MOI=25; B. Results of GFP virus infection with MOI=50; C. Results of GFP virus infection with MOI=100 |
